# Implementing real-time assessments of substance use cravings, triggers, and mood: a feasibility study with justice-involved populations

**DOI:** 10.1186/s40352-025-00372-2

**Published:** 2025-10-16

**Authors:** Ginnie Sawyer-Morris, McKenna Halverson, Kelly M. Maher, Steven B. Carswell, Michael S. Gordon

**Affiliations:** 1 Friends Research Institute, Inc, Baltimore, United States; 2 University of Delaware, Newark, United States; 3 MATClinics, Dundalk, MD, United States

**Keywords:** Ecological momentary assessment, Digital progress system, Justice-involved populations, Substance use treatment

## Abstract

**Background:**

The Daily Progress System (DPS) is a daily mobile health monitoring tool that allows clients with opioid use disorder to track substance use, recovery-related symptoms, and recovery support activities (e.g., 12-step groups) over a 24-hour period. This pilot study explored the feasibility of equipping the DPS with an ecological momentary assessment (EMA) feature.

**Methods:**

Thirty participants with justice involvement were recruited from an outpatient addiction treatment clinic in Maryland. Participants completed an integrated 14-day DPS + EMA protocol that assessed cravings, triggers, and mood three times per day (3–7 items; morning, afternoon, evening), and recovery-related symptoms and activities once per day (DPS, 10 items; evening). Financial incentives were provided for each survey completed. Descriptive statistics described sample characteristics and usage metrics (e.g., compliance). Friedman and Wilcoxon signed-rank tests were used to compare differences in non-normal compliance rates based on time of day and survey type.

**Results:**

Overall compliance with the EMA and DPS surveys was excellent (91.9%). No significant differences in compliance were observed based on time of day or survey type. In terms of acceptability, participants reported high overall satisfaction (*M*_*EMA*_ = 8.9/10; *M*_*DPS*_ = 9.5/10) and reported that the surveys were easy to understand (*M*_*EMA*_ = 4.9/5; *M*_*DPS*_ = 4.8/5). Qualitative feedback suggested that the EMA surveys helped participants identify patterns and glean additional insights into their mood, cravings, and triggers. Participants provided suggestions for future development, such as incorporating additional opportunities to report cravings and triggers outside of the primary delivery windows.

**Conclusion:**

These findings suggest that equipping the DPS with real-time assessments of cravings, triggers, and mood is feasible and acceptable among individuals with justice involvement who are engaged in outpatient treatment. Further research is needed with larger samples and over longer periods of time to evaluate the long-term benefits of this approach and its potential to enhance treatment adherence, patient engagement, and downstream recovery outcomes. However, these findings support the continued development and refinement of the DPS equipped with real-time assessments.

## Background

Substance use disorder (SUD) is a complex, chronic condition that continues to adversely impact individuals in the United States (Substance Abuse and Mental Health Services Administration [SAMHSA], 2025). However, the rates of individuals accessing treatment via telehealth has steadily increased since 2020. Of the 10.2 million individuals who received treatment for SUD in 2024, approximately 25% accessed it via telehealth (SAMHSA, 2025).

Over the last several years, treatment and monitoring of SUDs have expanded beyond traditional face-to-face formats to include digital health technologies (Marsch, 2020; McDonnell et al., 2021; Nesvåg & McKay, 2018; Sawyer-Morris et al., 2024). While studies have documented a substantially higher prevalence of SUDs among adults under justice supervision in the community (e.g., probation, parole; Gordon et al., 2011; Gryczynski et al., 2012; Morrison et al., 2024), digital health technologies are transforming how treatment services are accessed and delivered among this population (Leach et al., 2022; Sawyer-Morris et al., 2024; Wilde et al., 2023).

Digital health encompasses a growing range of products, including mobile health apps and web-based platforms for telehealth, which encourage clients to maintain healthy lifestyles and aid in treatment and recovery (Sawyer-Morris et al., 2024). By using mobile and wireless devices, these technologies mitigate barriers to traditional SUD services and allow for continuous monitoring and assessment of factors that may contribute to substance use.

Research demonstrates that patients have improved healthcare outcomes when their progress is routinely monitored (Crits-Christoph et al., 2012; Kidd et al., 2022). Routine outcome monitoring (ROM) is standard practice in the treatment of many chronic medical conditions (e.g., diabetes, hypertension; Ajjan et al., 2024; Angell et al., 2013); however, uptake of these practices in the substance use field has been less prevalent. In 2006, the Institute of Medicine (IOM) issued a report focused on quality care for SUD treatment and mental health, which outlined the need to develop monitoring devices that were both valid in assessing treatment progress and practical for routine use (Institute of Medicine (US) Committee on Crossing the Quality Chasm: Adaptation to Mental Health and Addictive Disorders, 2006). Since the release of this report, there has been a rapid proliferation of digital health monitoring tools developed to support substance use treatment, however, researchers must remain vigilant in enhancing, optimizing, and supporting the adoption of such tools as overdose death rates remain high with over 107,000 deaths recorded in 2023 (National Center for Health Statistics, 2024).

Traditionally, SUD treatment has been monitored with measures such as urine drug screens and patient attendance (Goodman et al., 2013). Although these indicators are useful for tracking patient progress, they do not sufficiently capture the complex array of factors that may predict substance use and recovery trajectories over time. For example, both internal (e.g., stress, negative affect) and external triggers (e.g., people, places, things) have been identified as predictors of substance use and recovery outcomes (Vafaie & Kober, 2022). For individuals with SUD, the ability to recognize the relationship between these factors and their risk for substance use may aid in recovery efforts. However, studies show that individuals vary in their ability to self-monitor and make these connections (Scott et al., 2018). Mobile health monitoring tools (e.g., Daily Progress Carswell et al., 2023), which allow patients to self-report these factors on a daily basis or in real-time, can help to fill these monitoring gaps and increase emotional and behavioral awareness among individuals in SUD treatment and recovery.

### Daily progress system

The Daily Progress System (DPS; Carswell et al., 2023) is a daily survey delivered via a web-based platform that assesses symptoms and social determinants of health relevant to SUD treatment and recovery. By generating a weekly summary report, which is shared with both clients and their counselors, the DPS helps clients increase their awareness of the contextual factors that may impact on their recovery. The DPS is designed to facilitate bi-directional communication and feedback between clients and their counselors outside of individual or group sessions, as well as reduce information deficits and improve treatment engagement and retention. The DPS assesses symptoms relevant to substance use treatment and recovery, such as alcohol and drug use, involvement in high-risk social situations, environmental triggers, and involvement in recovery-support activities (Carswell et al., 2023). Clients who participated in a 4-week feasibility trial using the DPS reported high levels of satisfaction with the platform and its utility as part of their SUD treatment (Carswell et al., 2023). Although findings from this feasibility study are promising, both clients and clinicians indicated that it would be useful to capture additional information about cravings (e.g., frequency of occurrence, patterns related to time of day) and triggers (e.g., people, places, and things). In the current study, we equipped the DPS with additional real-time, random assessments (i.e., brief surveys) of cravings and triggers using ecological momentary assessment (EMA).

### Ecological momentary assessment

EMA, a digital approach designed to capture and analyze real-time feedback from clients in their naturalistic environment, involves sending multiple signaled prompts throughout the day, often via text message or push notification (Shiffman et al., 2008; Singh & Björling, 2019). Prior substance use research demonstrates the utility of EMA for assessing clients’ cravings, triggers, moods, and behaviors, which are known to fluctuate throughout the day (Serre et al., 2015). Because these symptoms are dynamic, retrospective assessments, where clients are asked to recall symptoms over a 24-hour period, may be subject to recall bias and overrepresent recent and salient events. By capturing real-time data in natural settings, this approach addresses these threats and increases ecological validity (Stone & Shiffman, 2002). Thus, EMA may serve as a useful complement to the DPS to obtain a more comprehensive understanding of treatment progress among justice-involved individuals in recovery for SUD. However, justice-involved populations face unique barriers that may influence engagement with digital health interventions. For example, individuals navigating reentry may have limited smartphone access, lower digital literacy due to extended periods of incarceration, or competing supervision requirements or health-related social needs (e.g., housing instability), all of which can challenge sustained participation in digital research protocols (e.g., EMA; Reisdorf & DeCook, 2021). Given these factors, it is critical to assess whether this approach is feasible and appropriate for this population.

### Current study

This study explored the feasibility of equipping the DPS, a daily retrospective monitoring tool, with an EMA feature (i.e., brief signal-contingent assessments [surveys] of mood, cravings, and triggers sent at random times within predetermined windows). The EMA surveys were designed to complement the DPS daily retrospective survey (DPS Daily Survey; 10 items). Findings were used to guide the design of a larger randomized controlled trial (RCT) evaluating the effectiveness of the DPS among justice-involved individuals who are receiving treatment for opioid use disorder (OUD).

## Methods

### Participants

Participants were recruited via word-of-mouth and study flyers posted between March and May 2024 at an outpatient addiction treatment program with eight locations across Maryland. Research staff were invited to share information about the study during the first fifteen minutes of the participating clinic’s virtual weekly intensive outpatient (IOP) or outpatient (OP) treatment sessions. Individuals were eligible to participate if they: (1) were 18 years or older; (2) had a history of involvement with the justice system (e.g., arrest, incarceration; and 3) were actively enrolled in IOP/OP treatment. Interested individuals completed an online screener where they provided consent for research staff to verify their information with the participating clinic’s administrative data, for the purposes of identification and eligibility verification. Once verified, research staff scheduled a one-on-one virtual (zoom) enrollment appointment with each eligible participant. At the enrollment appointment, research staff reviewed study details per the WIRB-Copernicus Group (WCG^®^) IRB-approved protocol, and participants completed informed consent and a baseline survey. All participants were informed that participation was voluntary and were assured of their right to withdraw from the study at any point without penalty or impact on their treatment at the participating clinic.

**Fig. 1:**
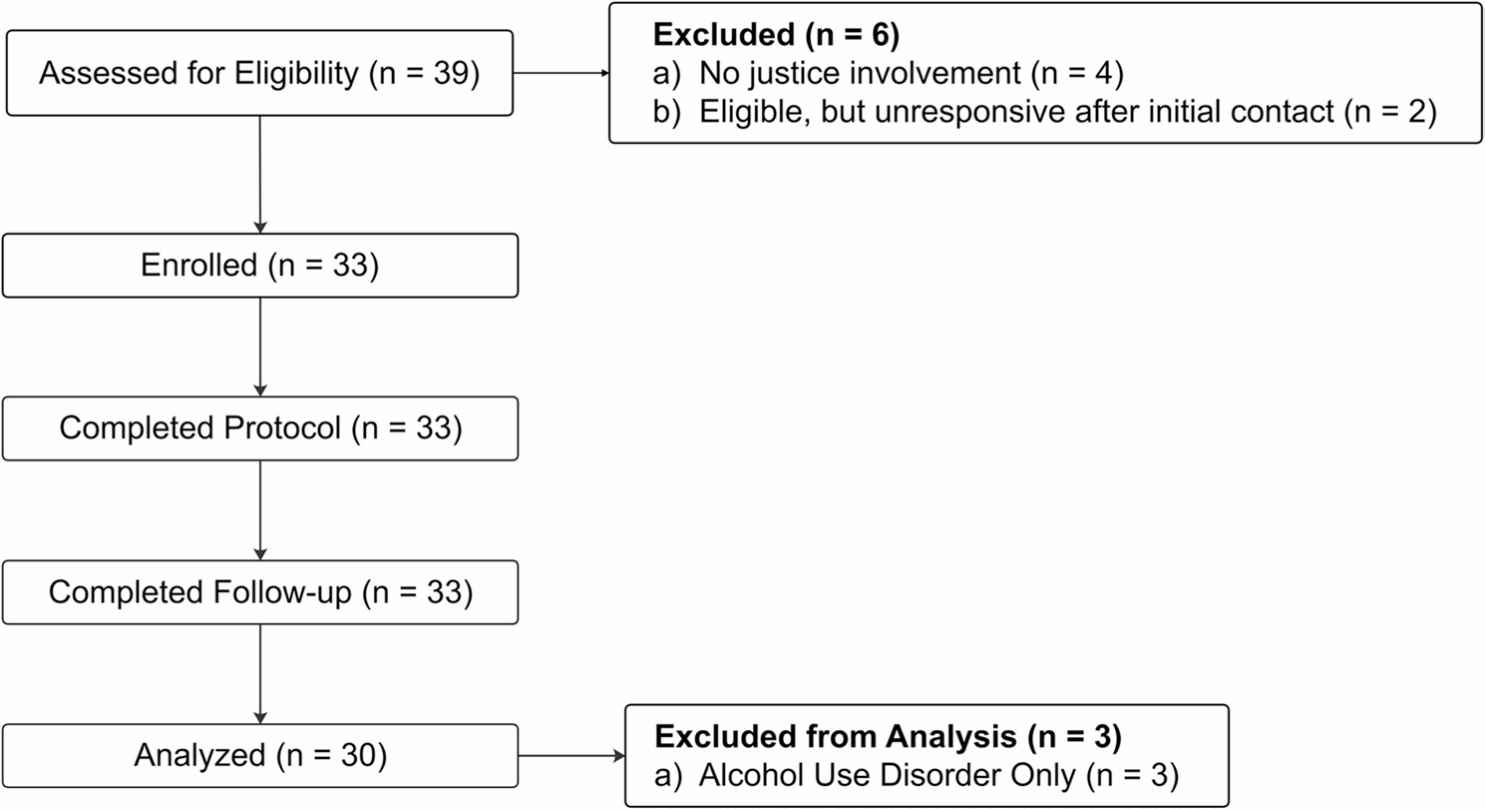
CONSORT Diagram

At the end of the appointment, research staff provided each participant with a virtual training on the EMA procedures. During the training, participants were sent a demo survey via text message that replicated the experience of receiving the actual EMA surveys. The 14-day EMA survey protocol was initiated the day after a participant’s enrollment appointment. Figure 1 illustrates the flow of participants through each stage of the study, from initial recruitment to final analysis.

The DPS platform and daily survey were developed and tailored for individuals with OUD or OUD in combination with other substance use disorders. Although the intervention was not designed for individuals with alcohol use disorder (AUD) only, these patients were permitted to participate in the study based on the potential for secondary benefit from self-monitoring and engagement. However, to ensure the generalizability of findings for RCT planning, AUD-only participants were excluded from the feasibility analysis (see Fig. 1). This approach allowed for a rigorous assessment of implementation feasibility with the target population, while still offering intervention access to individuals who otherwise met the eligibility criteria (i.e., 18 years or older, history of justice involvement, enrolled in IOP/OP treatment).

## Measures

### Baseline survey

Sociodemographic characteristics of participants were measured at baseline (see Table 2). The baseline survey also assessed substance use (lifetime and past 30 day use of opioids, cocaine, marijuana, and alcohol) and treatment history (number of lifetime treatment experiences for drugs, number of lifetime treatment experiences for alcohol), length of time in treatment (number of days), criminal justice involvement (number of times arrested, number of times incarcerated), and comfort-level with technology (0 = not at all comfortable, 10 = extremely comfortable).

### DPS daily survey and EMA surveys

The DPS (Carswell et al., 2023) is a web-based platform that administers a daily 10-item retrospective survey (i.e., DPS Daily Survey) to clients each evening. An EMA feature was added as an enhancement to the DPS platform. The EMA feature consisted of three brief signal-contingent surveys (three to seven items each) sent at random times within predetermined windows (morning, afternoon, evening). The EMA and DPS Daily Surveys are complementary. Therefore, the evening EMA survey (three to seven items each) was integrated with the DPS Daily Survey (DPS Daily Survey; 10 items) to simplify survey delivery and improve user experience.

#### DPS Daily Survey

The DPS Daily Survey (10 items; see Carswell et al., 2023 for a full description of the measure) assesses symptoms and activities relevant to substance use treatment and recovery across a 24-hour period. Domains assessed by the DPS Daily Survey include cravings, mood, involvement in high-risk situations, stress, alcohol and drug use, recovery support and wellness activities, and abstinence self-efficacy. Clients also have the option to provide open-ended feedback to their counselor via a free-text response field at the end of the survey.

While the DPS Daily Survey retrospectively assesses mood quality and craving frequency over a 24-hour period, it does not assess triggers. In a previous evaluation of the DPS platform (Carswell et al., 2023), participants suggested adding assessments of substance use cravings (e.g., severity, patterns related to time of day) and triggers (e.g., people, places, and things). Since mood, cravings, and triggers often fluctuate throughout the day, we chose to equip the DPS with an EMA feature, consisting of brief signal-contingent random assessments delivered three times daily (i.e., EMA surveys).

#### EMA Surveys

 The EMA surveys were designed to increase ecological validity and to serve as a complement to the DPS Daily Survey. Whereas the mood and cravings items on the DPS Daily Survey assess overall mood quality and craving frequency, the EMA surveys capture a contextualized, real-time snapshot of emotional state (checklist), mood (1–10), craving severity (0–10), and triggers (people, places, things; conditional on craving severity response > 0).

**Fig. 2:**
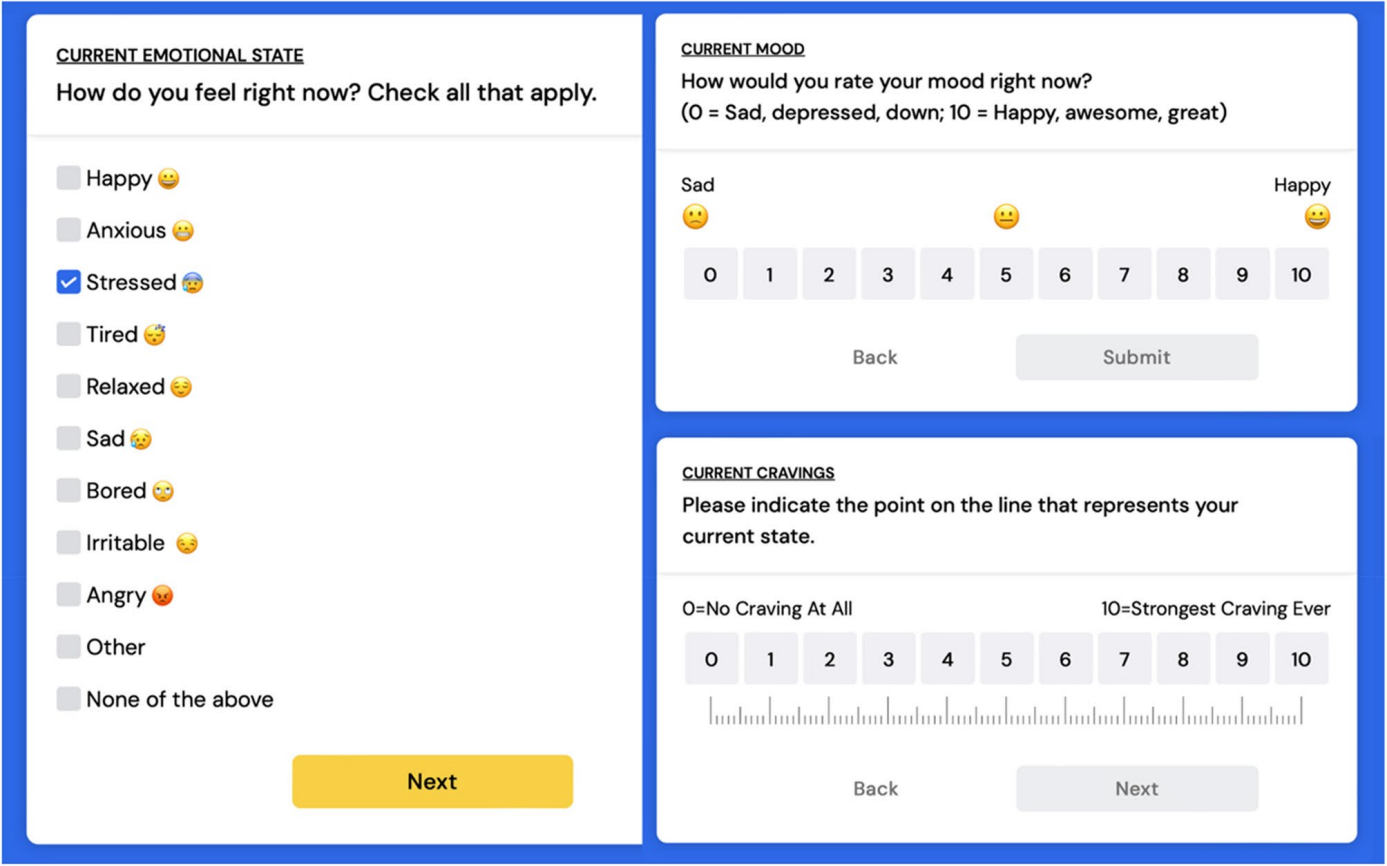
Example Items from EMA Survey: Emotional State, Mood, Cravings

Current emotional state was assessed using the following item, adapted from Linas et al. (2014): *“How do you feel right now? Check all that apply.”* Original response options from Linas et al. (2014) included “Happy,” “Stressed,” “Tired,” “Relaxed,” “Bored,” “Irritated,” and “None of the above.” The director of counseling at the participating clinic reviewed these options and recommended adding “Anxious,” “Sad,” “Angry,” and “Other” where, if selected, clients could write in a response (see Fig. 2 for final list of items).

##### Note

EMA = Ecological Momentary Assessment. The EMA surveys were delivered three times per day at random times within specified intervals (i.e., morning, afternoon, and evening). The evening EMA survey was delivered with the DPS daily 10-item retrospective survey.

Mood rating was assessed using the Ottawa Mood Scales (Cheng, 2011; Wong et al., 2021), a pictorial assessment where clients rate their mood on a scale of 1 to 10 (1 = “*Sad*,* depressed;*” 5 = *“In the middle*,* not happy or sad;”* and 10 = *“Happy*,* awesome*,* great”*).

Craving severity was measured using the Opioid Craving Visual Analog Scale (OC-VAS; Boyett et al., 2021), a widely used tool to measure the intensity of opioid cravings. Participants were asked to indicate the strength of their current craving on a 10-mm line, where 0 = *“No craving”* and 10 = “*Strongest craving ever*.”

In instances where participants reported craving severity greater than zero, a set of four follow-up questions about triggers displayed: *“****What*** *triggered your craving?” “****Who*** *were you with when you experienced the craving?” “****Where*** *were you when you experienced the craving?” and “****What were you doing*** *when you experienced the craving?”* We piloted two versions of response options to the trigger questions. During the first week of the protocol, participants provided open-ended responses; during the second week, participants were provided a checklist of response options and asked to “select all that apply.” The response options for the *“****What*** *triggered your craving?”* and *“****What were you doing*** *when you experienced the craving?”* questions were developed in collaboration with the director of counseling at the participating clinic. Example items for the ***“What****…”* question included, “Empty pill bottles,” “Special occasions and holidays,” and “Watching a movie where someone is using your drug of choice.” Example items for the ***“What were you doing****…”* question included, “Drinking alcohol,” “Planning/thinking”, and “Shopping.” The list of items for the *“****Who****…”* and “***Where****…”* trigger questions were adapted from Linas et al. (2015). Example response options for the ***“Who****…”* question included, “Alone,” “Family member,” and “A spouse/partner/significant other.” Response options for the ***“Where****…”* question included, “Another’s home,” “Car,” and “Store.” All checklists included an “Other” option where participants could write-in a response. During the follow-up survey, we asked participants which response type they preferred (i.e., checklist with write-in option vs. open-ended). The majority (83.3%) preferred the checklist where they could select all conditions that apply and write-in responses when needed. In our next study, we plan to further refine the checklist items using write-in responses from participants.

### Feasibility and acceptability measures

Feasibility was measured by assessing overall and individual-level compliance (survey completion rates) over the 14-day period. Acceptability of the EMA + DPS survey protocol was measured both quantitatively and qualitatively. Quantitative items included questions about *client satisfaction* (1 = extremely dissatisfied, 10 = extremely satisfied), *survey interpretability* (1 = not easy to understand, 5 = very easy to understand), and *survey interference with daily life* (1 = not a lot, 10 = extreme amount). Feasibility and acceptability questions were asked separately for the EMA and DPS Daily surveys. Previous EMA studies have demonstrated that financial incentives are positively related to compliance (Howard & Lamb, 2024; Ottenstein & Werner, 2022; Wrzus & Neubauer, 2023). To assess the impact of financial incentives on motivation in this study, participants were asked to report the degree to which they agreed with the following statement (1 = Strongly Disagree, 5 = Strongly Agree): “Even if I did not receive payment, I would have completed the same number of surveys if I knew the information would be used to inform my substance use treatment.” Qualitative acceptability items included open-ended questions exploring client feedback about the EMA and DPS surveys (e.g., likes/dislikes, ways to improve the surveys), user experience with the platform, and the impact of financial incentives on an individual’s motivation to complete the surveys.

### A priori codes

Acceptability and usability were assessed qualitatively via open-ended questions and analyzed using a deductive approach (i.e., a priori coding; see Bingham, 2023). Table 1 outlines the a priori codes, their definitions, and conceptual examples.

**Table 1:** A priori codes

Code	Definition	Example
*Acceptability*	Focuses on overall satisfaction and whether the intervention is perceived as fitting the needs and context of the target population (i.e., justice-involved patients in intensive outpatient/outpatient treatment).	Satisfaction
*Usability/* *User Experience*	Focuses on specific user needs of target population, factors that influence user experience. For example, perception of form/function of the interface, identifying technical or design flaws.	Ease of use
*Suggestions for* *Future Development*	Recommendations, or critiques intended to improve, modify, or expand the intervention, program, or tool. This includes areas for improvement, feature requests, etc.	Suggestions for improvement
*Incentives*	Perceptions of incentives, including role of incentives in engagement with surveys.	Mentions of motivation in relation to incentives

### Procedures

**Fig. 3:**
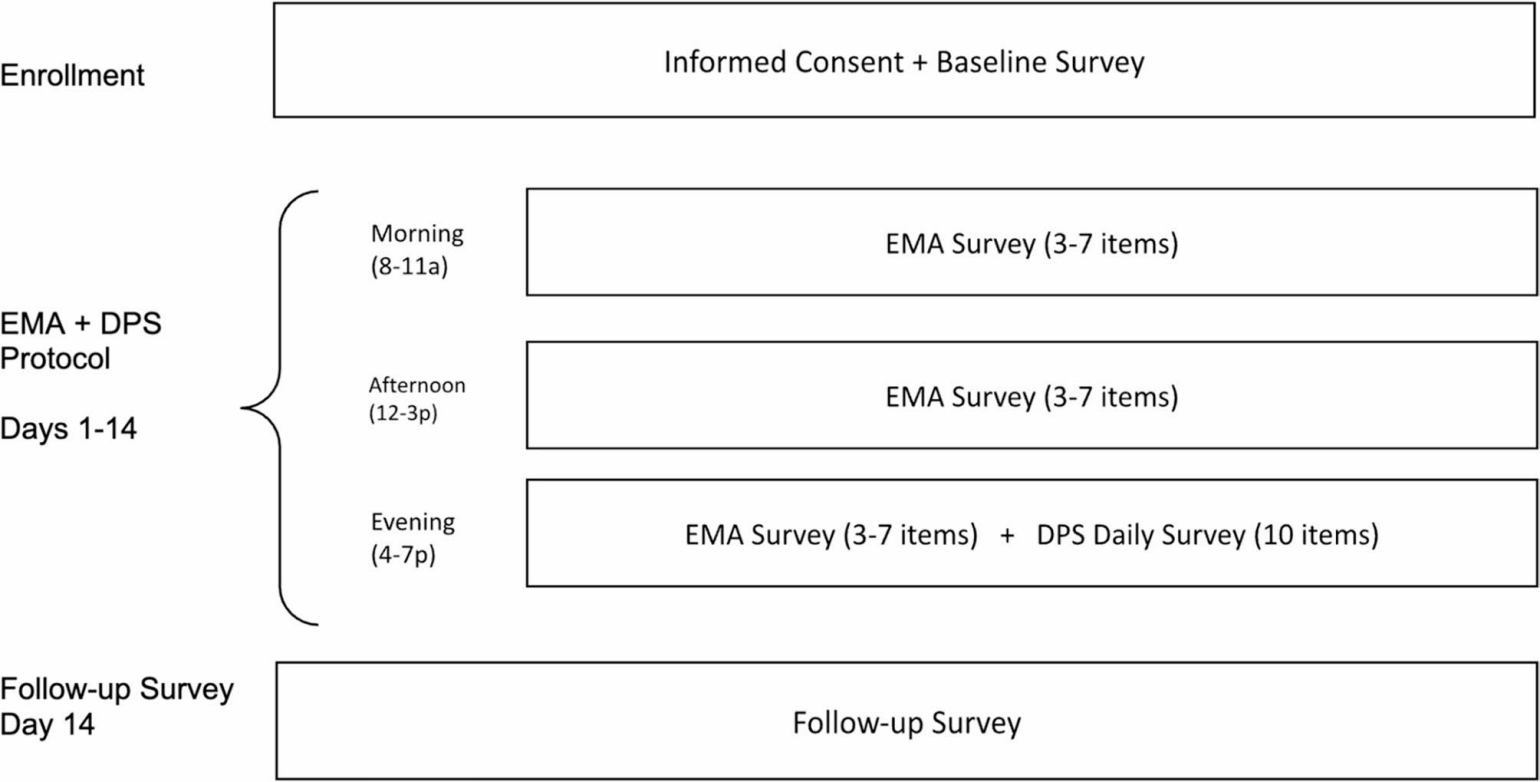
EMA and DPS Daily Survey Delivery Schedule

The 14-day protocol (see Fig. 3) was implemented using semi-random sampling, where participants received signal-contingent prompts via text to complete three surveys delivered at random times within three predetermined windows (morning: 8-11a; afternoon: 12-3p; evening: 4-7p). If participants did not respond to any given prompt within 15 min of receiving the survey, they were sent a reminder. Participants received one reminder per assessment (i.e., up to three total reminders per day). The morning and afternoon surveys included only the EMA items (three to seven items; three items if craving severity = 0, seven items if craving severity > 0), and the evening survey included 13–17 items (three to seven EMA items; 10 DPS Daily Survey items). The original DPS platform administers a daily retrospective survey (DPS Daily Survey; 10 items) each evening. In the current study, we integrated the DPS Daily Survey (10 items) with the EMA survey (3–7 items) to capture a comprehensive end-of-day profile that included both real-time assessments and retrospective data from the prior 24 h (see Fig. 3). Our study goal was to determine whether participants would continue to complete the daily retrospective survey (DPS Daily Survey) in addition to the newly added EMA surveys.

While delivered as one survey, the evening EMA and evening DPS Daily surveys were considered two distinct assessments for the purposes of compensation and compliance. The integrated evening survey was delivered between 4-7p. If completed before 8p, the evening survey included both the EMA items and the DPS Daily Survey items. If participants completed the evening survey after 8p, it included only the DPS Daily Survey items (see additional information below). Participants also completed a follow-up survey after study completion, which assessed survey satisfaction, as well as feedback about the usability of the platform and its various features. Participant incentives included $55 for completing the baseline survey, $5-$10 for each survey completed ($5 for morning/afternoon EMA surveys, $10 for evening EMA/DPS Daily surveys), and $35 for completing the follow-up survey. If participants completed > 50% of the surveys in one week, they were eligible for a bonus payment ($10); the more surveys they completed, the higher their weekly bonus (up to $35 for completing 95% of surveys). Finally, participants were given a smartphone to use and keep upon study completion, which is common practice in mHealth substance use treatment studies (see Gustafson et al., 2024; Scott et al., 2017). Or, if they already owned a smartphone, they were paid a $150 stipend to cover the cost of their data plan for the duration of the study. We offered the stipend option to ensure that individuals who opted to use their own phone received a comparable benefit to those who opted to receive a new phone. The stipend option is intended to alleviate the barriers that would otherwise be mitigated if the client had chosen to use and keep the study phone, while avoiding the hassle of changing their phone number and data plan. All participant incentives were paid electronically via Cash App (93.3%) or by mailing a physical gift card (3.3%).

#### Note

EMA = Ecological Momentary Assessment. DPS = Daily Progress System. All surveys were sent at random times within the morning (8a-11a), afternoon (12-3p), and evening (4-7p) windows.

### Analysis

Descriptive statistics were calculated for demographic variables and usage metrics (e.g., overall compliance, compliance by time of day). Group comparisons with non-normally distributed feasibility variables (e.g., compliance by time of day, compliance by survey type) were conducted using the Friedman test for three or more related groups and the Wilcoxon signed-rank test for two related groups, non-parametric alternatives to the repeated measures ANOVA and paired-samples t-test, respectively (Friedman, 1937; Wilcoxon, 1945). Group comparisons with non-normally distributed acceptability variables (e.g., client satisfaction, survey interpretability) were conducted using the Wilcoxon signed-rank test, a non-parametric alternative to the paired samples t-test (Wilcoxon, 1945). Qualitative acceptability data from open-ended questions were analyzed using a deductive approach (i.e., a priori coding; (Bingham, 2023; Crabtree & Miller, 1999). We used a priori coding (Bingham, 2023) to identify key themes related to acceptability and user experience. Quantitative analyses were conducted using Stata 18.0 (StataCorp, 2023) and qualitative analyses were conducted using Atlas.ti (Lumivero, 2024).

## Results

### Participants

Between April and June 2024, a total of 30 participants completed the study. Participants were majority White (76.7%), female (60%), reported an average household income of $25,000 or less (73.3%), and ranged in age between 25 and 65 years (*M* = 39.2, *SD* = 9.9). At baseline, 50% of participants reported opioids as their primary substance of use, while 40% reported multiple substances, and 10% reported cocaine. In terms of substance use treatment, the average number of lifetime substance use treatment episodes among participants was 4.4 (*SD* = 3.9). All individuals in the sample had a lifetime history of justice involvement, 23% of participants were referred to treatment through the justice system (e.g., drug court, probation, parole) and 36.7% were currently under community supervision at the time of enrollment. Table 2 describes the sample in terms of sociodemographic characteristics.

**Table 2:** Sociodemographic characteristics

Characteristic	*N* = 30^*1*^
Age at Enrollment	39.2 (9.9)
Race/Ethnicity †	
White, non-Hispanic	23 (76.7%)
African American/Black, non-Hispanic	4 (13.3%)
Hispanic	2 (6.7%)
Multiracial, non-Hispanic	1 (3.3%)
Gender †	
Women	18 (60.0%)
Men	12 (40.0%)
Education Level †	
Middle School (Jr. High School or less)	2 (6.7%)
Some High School, No Diploma/GED	8 (26.7%)
High School Graduate, GED or Equivalent	14 (46.7%)
Some College, No Degree	4 (13.3%)
Associate Degree (2-year): Occupational	1 (3.3%)
Bachelor’s Degree (Example: BA, BS)	1 (3.3%)
Income †	
Less than $5,000	11 (36.7%)
$5,000 - $9,999	4 (13.3%)
$10,000 - $24,999	7 (23.3%)
$25,000 - $49,999	5 (16.7%)
$50,000 or More	1 (3.3%)
Don’t know	2 (6.7%)
Employment Status †	
Working Now	11 (36.7%)
Looking For Work, Unemployed	10 (33.3%)
Keeping House	3 (10.0%)
Disabled, Permanently or Temporarily	2 (6.7%)
Retired	1 (3.3%)
Other	2 (6.7%)
* Missing*	1 (3.3%)
Type of Insurance Coverage †	
Type of Health Insurance: Medicaid	26 (86.7%)
Type of Health Insurance: Medicare	1 (3.3%)
Type of Health Insurance: State-Sponsored Health Plan	1 (3.3%)
Type of Health Insurance: Private Health Insurance	1 (3.3%)
Type of Health Insurance: State-Sponsored Health Plan	1 (3.3%)
Risk for Household Food Insecurity ‡	24 (80.0%)
Housing Status: Homeless ‡	5 (16.7%)

^*1*^ Mean (SD); n (%)

† Categorical Variable

‡ Dummy Variables

## Feasibility

### Compliance rates

No individuals discontinued the study, therefore, attrition rates were not assessed. Average overall compliance was 91.9% across all individuals. Across the different delivery windows (morning, afternoon, evening), compliance was highest for evening surveys (92.8%), followed by the afternoon (91.7%) and morning (91.2%) surveys. Differences in compliance rates based on delivery window (i.e., time of day) were examined using a Friedman test. No significant differences were observed across the morning, afternoon, and evening surveys (*p* =.634), which suggests that participants maintained consistently high compliance rates regardless of the time of day the surveys were administered. Differences in survey type (i.e., average compliance for EMA only surveys [morning, afternoon] vs. integrated DPS Daily Survey + EMA survey [evening]) were examined using a Wilcoxon signed-rank test. Average compliance was lower for the EMA only surveys (91.4%), relative to the integrated DPS Daily Survey + EMA survey (92.8%), though not significantly (exact *p* =.342).

## Acceptability

### Satisfaction and usability ratings

Participants reported high satisfaction with both the EMA surveys (*M* = 8.9/10, *SD* = 1.6) and DPS Daily Surveys (*M* = 9.5/10, *SD* = 1.1). The surveys were rated as very easy to understand for both EMA (*M* = 4.9/5, *SD* = 0.4) and DPS (*M* = 4.8/5, *SD* = 0.4). A series of Wilcoxon signed-rank tests indicated no statistically significant differences between the EMA and DPS Daily surveys in terms of overall satisfaction (*p* =.093) and survey item interpretability (*p* =.623). Survey interference with daily life was reported as minimal for the surveys overall (*M* = 0.1/10, *SD* = 0.4).

### Qualitative themes

Open-ended responses were coded independently by two researchers from the study team. Data were imported into Atlas.ti and sorted by survey question. Keywords from text responses were selected in alignment with a priori themes and used to code the responses. A priori codes for deductive analysis included “Acceptability,” “Usability/User Experience,” “Suggestions for Future Development,” and “Incentives” (see Table 1). Participants found the EMA + DPS protocol to be acceptable, reported positive user experience feedback, provided suggestions for changes and improvements, and offered feedback on how the incentives impacted their motivation to complete surveys.

#### Acceptability

All participants agreed on acceptability:

I liked it because it put things in perspective. By the time I was done the survey I realized more about my addiction than [I knew] before.

I like it, helped me get through my day.

I love it, something else to do during the days I don’t have my groups.

#### Usability/User Experience

Participants similarly agreed on usability, noting that the surveys were simple, convenient, easy to understand, and helpful as a self-reflection tool:

The [EMA] surveys were short and simple, but if you were having a craving, there was a lot of opportunity to explain why.

I liked the self-reflection part of it, I was able to understand how I’m feeling and when and why better after this. No dislikes.

It was super easy and self-explanatory.

#### Suggestions for Future Development

Participants also made suggestions for future development related to both content and user experience. Content suggestions included adding the following questions to the DPS Daily surveys:

How long have you been clean or sober?

[What are] your overall thoughts about your addiction and current recovery today?

User experience suggestions included adding variety by shuffling questions around, incorporating additional features, and integrating coping strategies and tips for when cravings were reported. When asked about overall suggestions, participants said:

… if u said u [were] having a bad day, maybe give a opportunity to the person to talk to someone or get them help if they wanted it.

Just move some of the questions around.

The DPS includes a weekly summary report, which was not included as part of this study. When asked if adding a weekly summary report would be useful, participants reflected:

Identifying patterns in my behavior was something I actually experienced during the survey, so yes, to see a weekly report would also be a positive tool to identify behaviors.

“Yes. I could study the summary to get an idea about how to improve my recovery program.”

Participants also suggested adding more questions and integrating additional opportunities to report cravings, mood, and triggers outside of the three primary delivery windows. When asked what could be improved about the surveys, participants responded:

More questions on the morning and afternoon surveys and maybe a chance to reply more often about mood and cravings throughout the day and even overnight.

Maybe just to be able to enter triggers throughout the day and a way to enter the time and place and what occurred.

#### Incentives

Overall, participants agreed that they would complete the same number of surveys if they were not being paid (*M* = 4/5, *SD* = 1.4), however, qualitative feedback provided additional insights. Representative examples of participant feedback are listed below based on level of agreement.

When asked whether they would complete the same number of surveys without being paid, participants with lower levels of agreement (Strongly Disagree to Disagree) indicated:

“I probably would have forgot.” (Strongly Disagree).

“It’s nice to give information that will help yourself and others, but honestly in this case the money was the greatest motivator. Just volunteering the info wouldn’t get as- often responses.” (Disagree).

A participant with neutral agreement stated:

“I am not positive or sure on this. Don’t get me wrong [money] helped since [I’m] not working but I’d prolly participate.” (Neither Disagree or Agree).

Participants with higher levels of agreement (Agree to Strongly Agree) said:

“I would [be] willing to participate in [the] same survey with no compensation but not sure I would have been as motivated to continue, especially the last week when it really starts dragging on.” (Agree).

“Even though the money motivated me I would have still done it after I seen how the first one went because it… helped me be more knowledgeable about my craving.” (Strongly Agree).

## Discussion

This study examined the feasibility and acceptability of implementing an EMA enhancement to an existing mobile health monitoring tool (i.e., DPS) among justice-involved individuals in outpatient substance use disorder treatment. High compliance and positive user experience feedback suggest that it is feasible to equip the DPS with incentivized real-time assessments of cravings, triggers, and mood with this population.

### Feasibility

#### Compliance

Average compliance over the 14-day study was 91.9%. While there is no established threshold for adequate compliance in EMA studies of substance use, rates considered to be “good,” “adequate,” or “excellent” range between 70 and 90% (Jones et al., 2019; Shiffman, 2009; Tonkin et al., 2023). In their meta-analysis of EMA studies with substance-using populations, Jones et al. (2018) used a benchmark minimum of 80% compliance, and found that the pooled compliance rate of EMA studies with substance-using populations between 1998 and 2017 fell below this threshold (75.6% across studies; Jones et al., 2019). A recent EMA study with undergraduate alcohol drinkers employed categorical cut points to classify compliance trends; participants were designated as “*super responders*” if they responded to more than 90% of surveys, “*good*” if they responded to 75% to 89% of surveys, “*adequate*” if they responded to 50% to 74% of surveys, and “*poor*” if they responded to less than 50% of surveys (Howard & Lamb, 2024, p. 281). Overall compliance in the current study, at 91.9%, meets or exceeds these benchmarks.

The higher compliance rates in our study may be attributed to several factors, including the use of financial incentives, the small sample size (*N* = 30 participants), which allowed for frequent participant contact, and the relatively short duration of the study (i.e., 14 days). Other literature has shown that compliance rates tend to decline as studies progress over time, varying as a function of assessment period (i.e., duration of study; Stone & Shiffman, 2002). However, this pattern was not observed in the current trial. This may have been due to the shorter length of the study.

#### Compliance based on time of day and survey type

No significant differences were observed in compliance rates based on time of day (i.e., delivery window) or survey type (EMA only surveys vs. integrated DPS Daily Survey + EMA survey). Despite the integrated DPS Daily + EMA Survey containing 10 more items than the morning and afternoon EMA only surveys, the evening integrated survey registered the highest rates of compliance. The slightly higher compliance rates for the evening surveys (92.8%), relative to the morning (91.2%) and afternoon (91.7%) surveys, may have been related to the additional compensation participants received. The incentive was increased for the evening survey to adjust for the added time it took participants to complete the additional items. This decision is supported by the broader EMA literature, which suggests that the provision of financial incentives has a significant and positive influence on compliance (Howard & Lamb, 2024; Ottenstein & Werner, 2022; Wrzus & Neubauer, 2023). In a recent meta-analysis of EMA studies across research fields, compliance rates were significantly higher in studies that provided financial incentives; other factors, like design and sample characteristics, had few effects (Wrzus & Neubauer, 2023). Similar results (i.e., improved compliance) were observed in contingency management interventions, where participants received an incentive contingent upon some type of abstinence-related behavior (e.g., negative urine toxicology screens; (Petry et al., 2005, 2006), demonstrating both short-term (e.g., 12-week; Petry et al., 2005) and long-term (e.g., 1-year post treatment; Ginley et al., 2021) efficacy. Depending on the design, EMA studies that provide financial incentives may function as a type of contingency management intervention. Future studies could consider ways to integrate these two approaches, leveraging the motivational power of incentives from contingency management and the real-time behavioral tracking capabilities of EMA, to develop interventions that not only maximize adherence but also foster increased behavioral awareness and sustained engagement in recovery programming.

### Acceptability

The high acceptability ratings and positive user experience feedback in our study suggest that participants with justice involvement who were engaged in outpatient treatment were willing to use the DPS equipped with an EMA feature. Qualitative findings further suggest that the EMA surveys helped promote participants’ awareness of mood, substance use cravings, and triggers. The act of repeatedly assessing one’s mood and cravings may promote personal insight into patterns that can support readiness for change. In the precontemplation and contemplation stages of the transtheoretical model (i.e., stages of change model; Prochaska & Diclemente, 1986; Sutton, 2001), which is often used to inform treatment in clinical settings, becoming conscious of one’s current patterns of behavior plays a key role in building readiness for change (SAMHSA, 2019). Mobile health monitoring tools, like the DPS equipped with real-time assessments, could be particularly useful in this regard, especially for individuals who were referred to treatment by an external entity (e.g., justice system) and who may need additional resources to build readiness. Future studies could explore the potential mediating or moderating effect of awareness on treatment engagement, examining whether, how, and for whom behavioral and emotional awareness, accumulated through real-time assessments, might contribute to improved outcomes in treatment engagement and overdose prevention.

## Strengths & limitations

Despite this study’s strengths, there are several key limitations that are important to note. First, this was a two-week feasibility study with a small sample size (*N* = 30) in which participants were recruited from one outpatient program. Therefore, additional studies over longer timeframes, with larger sample sizes, and across multiple sites are warranted. Additionally, participants in our study received incentives for completing the EMA and the DPS Daily suveys, so it is unclear whether our results would translate to real-world settings in which incentives were not distributed. Furthermore, the DPS was designed to be a client- and clinician-facing platform, where clients share their survey responses with clinicians to help facilitate therapeutic alliance. However, neither the DPS nor the EMA survey results were shared with clinicians in this study, which may have influenced how participants responded to the items (i.e., possibly being more forthcoming in their responses).

### Duration of study

The length of EMA studies vary throughout the literature. Reviews of EMA studies have shown that compliance rates tend to decrease as a function of study length (Jones et al., 2019; Stone & Shiffman, 2002). Two weeks (i.e., duration of present study) is a common timeframe for a feasibility trial, however, studies over longer periods often report declines in compliance (Jones et al., 2019). This study explored the feasibility of adding an EMA enhancement to the DPS platform. Because the DPS is a daily retrospective monitoring tool, it is critical that added features or enhancements not detract from engagement in the DPS daily survey. While we observed improved compliance in the evening survey over the two-week period, it is unclear whether our compliance rates would have sustained over longer periods of time (> two weeks). But a better question to ask might be, should we expect them to? The discrepancies in compliance between shorter (one to two weeks) and longer (≥ three weeks) EMA studies beg to question whether EMA should be deployed as a monitoring tool over long periods of time. Or should EMA interventions be best delivered in shorter bursts (e.g., two weeks) every three or so months to reliably capture change over time while mitigating participant burden? Future studies could consider exploring the efficacy of two-week EMA bursts (Helle et al., 2023) in promoting treatment compliance or in engaging (or re-engaging) difficult-to-reach clients. Alternatively, the EMA protocol could be used as a springboard for the DPS to promote sustained engagement. For example, the EMA surveys could be deployed with the DPS Daily surveys for two weeks, as in the present study, and then removed the third week so that participants are only completing the once daily DPS survey alongside their outpatient treatment.

### Incentives

The present study provided financial incentives to participants for completing surveys over a two-week period. It is unclear whether the same participants would have continued completing the surveys with the same level of compliance (i.e., > 90%) if financial incentives were removed during the study or not provided at all. Stakeholders (e.g., clinic administrators, policy makers, payers) have conveyed concern about the feasibility (i.e., cost-benefit ratio, reimbursement) of providing financial incentives (Petry et al., 2017). However, the growing use of contingency management in SUD treatment settings demonstrates that incentives can be both effective and cost-efficient in improving treatment adherence and reducing substance use (Coughlin et al., 2023; Olmstead & Petry, 2009; Petry et al., 2017). In contingency management interventions, incentives are provided for a predetermined amount of time, which can range from several weeks to several months (Coughlin et al., 2023). Future evaluations of the DPS equipped with EMA could randomize participants to different pay schedules used in contingency management (e.g., escalating schedule, escalating schedule with reset, constant schedule; see Coughlin et al., 2023) to compare differences in compliance for the two-week protocol.

### Building rapport with participants

Because this was a relatively small sample (*N* = 30), research staff were able to consistently engage with participants to provide technical support, answer questions when needed, and complete a midpoint check-in to offer words of encouragement. Consistent contact with participants, while effective in building rapport, may prove difficult in larger studies or not feasible if the task falls on counselors. Future implementation studies with the DPS equipped with EMA may consider partnering with peer recovery support specialists as part of the implementation protocol to build in additional connection and support for clients. This could be key in early stages of implementation when participants are still learning how to engage with the platform.

### Anonymity

The DPS is designed to facilitate bi-directional communication and feedback between clients and their counselors outside of individual or group sessions, as well as to reduce information deficits and improve treatment engagement and retention. However, engaging the counselors was unfortunately outside the scope of the current study, which was to test the feasibility of equipping the DPS with an EMA feature (i.e., brief signal-contingent assessments of mood, cravings, and triggers sent at random times within predetermined windows). We wanted to determine that the protocol was feasible with participants (i.e., patients engaged in IOP/OP) before engaging the counselors. There is a possibility that participants may have responded differently since the survey results were not being shared with their counselors. When participants were asked if they would be open to sharing their survey responses with counselors, a little over half of participants reported they would be comfortable doing so to help inform their treatment. Others preferred anonymity, indicating that the surveys provided a space where they could be vulnerable and honest about situations they might not feel comfortable discussing in group. Anonymity has long served as a pillar of recovery, offering a protective space for people to heal without fear of judgment or exposure. Findings from a recent participatory design study that engaged individuals in early recovery as co-designers of recovery support technology indicated that managing stigma, privacy, personal and group anonymity, trust, and personal safety were all key design considerations (Schmitt & Yarosh, 2018). The authors recommended that future recovery technology efforts establish anonymity and safety practices that can be continually reinforced through socio-technical means (Schmitt & Yarosh, 2018). Future client- and clinician-facing health monitoring tools could empower users by incorporating HIPAA-compliant privacy settings. These settings could allow clients the choice to use the tools solely for self-monitoring or to share results with counselors when they feel secure, thereby balancing privacy with treatment engagement.

## Conclusions

In conclusion, we found that implementing the DPS with an integrated EMA enhancement is feasible among justice-involved individuals engaged in outpatient treatment for substance use disorders. The high compliance rate (91.9%) in this study suggests that implementation of incentivized digital health monitoring interventions among this population is promising. Integrating real-time (EMA) and daily retrospective monitoring (DPS) provides a more comprehensive snapshot of individuals’ experiences in recovery, potentially offering valuable insights for both clients and clinicians. However, the relative value of these integrated modalities may depend on an individual’s stage of recovery and the intensity of current symptoms. For example, DPS + EMA may be particularly beneficial to individuals in early treatment, as part of establishing consistent routines and self-monitoring habits to support behavioral stability and self-regulation (Laudet & White, 2008; Witkiewitz et al., 2019). Whereas the DPS alone may suffice for individuals further along in recovery who experience less frequent or intense cravings. While further research is needed to establish the long-term benefits and generalizability of this integrated approach, these findings support the continued development and refinement of the DPS equipped with real-time assessments.

## Data Availability

The data supporting the findings of this study are not publicly available due to the potential for participant identification. Researchers interested in accessing the data may contact the corresponding author to discuss potential data sharing options under strict confidentiality agreements, ensuring participant privacy is protected at all times.
